# Weight Changes following the Diagnosis of Type 2 Diabetes: The Impact of Recent and Past Weight History before Diagnosis. Results from the Danish Diabetes Care in General Practice (DCGP) Study

**DOI:** 10.1371/journal.pone.0122219

**Published:** 2015-04-15

**Authors:** Niels de Fine Olivarius, Volkert Dirk Siersma, Rasmus Køster-Rasmussen, Berit Lilienthal Heitmann, Frans Boch Waldorff

**Affiliations:** 1 The Research Unit for General Practice and Section of General Practice, Department of Public Health, University of Copenhagen, Copenhagen, Denmark; 2 Institute of Preventive Medicine, Research Unit for Dietary Studies, Frederiksberg Hospital, Frederiksberg, Denmark; Johns Hopkins Bloomberg School of Public Health, UNITED STATES

## Abstract

**Aims:**

The association between recent and more distant weight changes before and after the diagnosis of type 2 diabetes has been little researched. The aim of this study is to determine the influence of patients’ weight history before diabetes diagnosis on the observed 6-year weight changes after diagnosis.

**Methods:**

A clinical cohort study combined with self-reported past weight history. In total 885 patients aged ≥40 years and newly diagnosed with clinical type 2 diabetes were included. Body weight was measured immediately after diabetes diagnosis and again at the 6-year follow up examination (median, 5.7 years). At diagnosis patients reported their weight 1 year and 10 years previously, and also at the age of 20. Multivariate linear regression analyses controlled for 20 baseline patient characteristics.

**Results:**

The median (interquartile range) age at diagnosis was 63.2 (53.9; 71.4) years. Median body weight was 80.0 (72.0; 90.0) kg 10 years before diagnosis, 85.0 (75.0; 95.0) kg 1 year before diagnosis, 82.4 (72.0; 94.0) kg at diagnosis, and 80.0 (70.0; 91.1) kg at 6-year follow up. Each kg of weight gain during the year preceding the diagnosis was associated with a weight change (95% CI) of -0.20 (-0.28; -0.13) kg during the follow up period. In all models age and body mass index at diagnosis predicted future weight changes, while the weight at age 20 (-0.01 (-0.06; 0.03) kg/kg), and the weight change from 10 years to 1 year before diagnosis (-0.01 (-0.06; 0.04) kg/kg), did not predict weight change after diagnosis.

**Conclusions:**

During the first on average 5.7 years after diagnosis of type 2 diabetes, patients generally follow a course of declining average weight, and these weight developments are related primarily to recent weight changes, body mass index, and age, but not to the more distant weight history.

## Introduction

During the past 40 years the prevalence of overweight and obesity as well as type 2 diabetes has increased worldwide [1], especially among the relatively young [2], and the association between weight gain during most periods of life and later development of diabetes is strong and well documented [3]. In type 2 diabetes weight loss is a fundamental treatment modality which is accompanied by improved cardiovascular risk factors and less use of medication [4]. However, weight loss has been reported to be even more difficult for people with type 2 diabetes than for those without diabetes [5]. On the other hand, patients are often more motivated to lose weight immediately after diabetes diagnosis [6], and this treatment window is often used in clinical practice. Until now, the relation between long-term weight changes both before and after diabetes diagnosis has only been studied systematically in Pima Indians, a population susceptible to obesity [7].

We studied the influence of patients’ weight history before diabetes diagnosis on the observed weight changes during the first 6 years after diagnosis in a population-based sample of patients newly diagnosed with clinical type 2 diabetes.

## Patients and Methods

### Study population and setting

**Fig 1:**
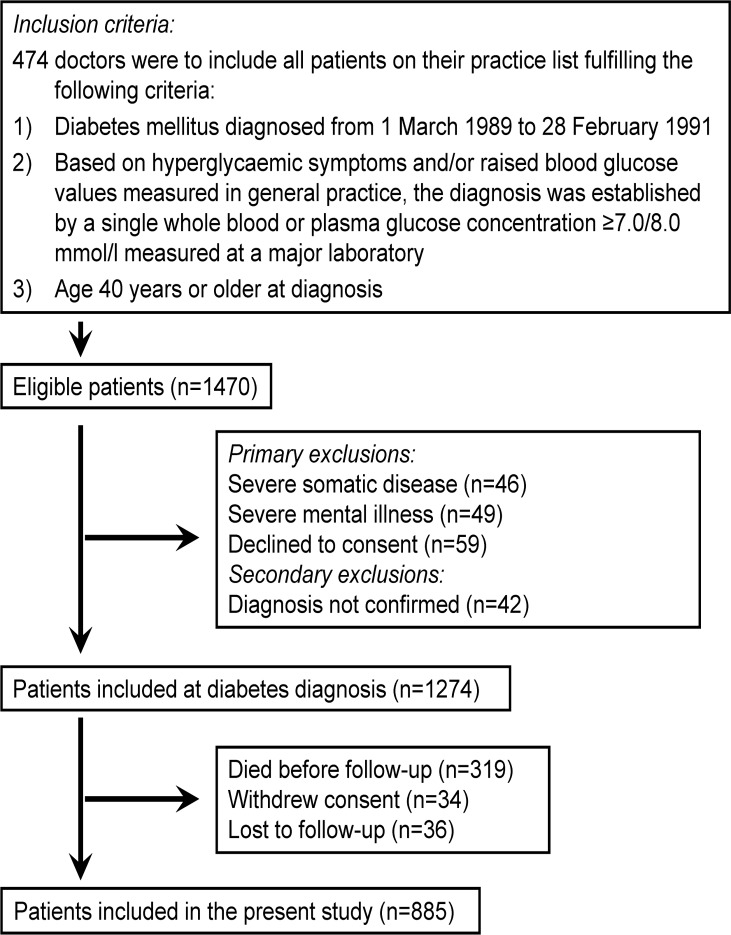
Patient flow.

The Diabetes Care in General Practice (DCGP) study was a pragmatic, open, controlled trial with randomisation to structured personal care or routine care [8]. In this study, 474 general practitioners included 1,274 patients with newly diagnosed diabetes based on specific diagnostic criteria (Fig 1). Of the 1,274 patients, 1,263 (99.1%) were of Western European descent. A small number started insulin treatment within 180 days of diagnosis, so 97.6% of the patients were considered to have type 2 diabetes [8]. The final study population comprises the 885 people with diabetes who survived to and carried through the 6-year follow up examination (Fig 1).

The intervention included regular follow up and individualized goal setting supported by prompting of doctors, clinical guidelines, feedback, and continuing medical education [8]. It was suggested to the intervention doctors that they recommend increased physical exercise and simple dietary rules: increase complex carbohydrate to at least 50% of the diet, and in particular increase water soluble fibre; reduce fat content to a maximum of 30%; reduce alcohol intake, and eat 5–6 meals a day [9]. After 6 years of intervention there was no statistically significant difference in body weight between the two randomisation arms [8].

### Ethics Statement

This study was approved by the research ethics committee of Copenhagen and Frederiksberg (V.100.869/87), and all patients gave oral informed consent to their general practitioner. Written informed consent was not customary in Denmark when the DCGP study was initiated.

### Assessments

Immediately after diabetes diagnosis, the general practitioners in our study did a structured clinical examination of each patient [8]. The time from the day of diagnosis until measurement of weight at diabetes diagnosis (W_diag_) was ≤30 days in 79.1% of the patients, and ≤60 days in 90.5%. Retinopathy was assessed by practising ophthalmologists. In questionnaires completed at diabetes diagnosis, patients gave information about whether they lived alone, their education, familial disposition to diabetes, smoking habits, leisure time physical activity, angina pectoris, intermittent claudication, and former or present cancer. Leisure time physical activity was assessed as the average physical activity level during the last year, graded in four levels, where 1 was hard training multiple times a week and 4 was inactive behaviour [10,11]. At diagnosis the patients were also asked to answer questions about their recollection of what their weight was 1 year (W_-1_) and 10 years (W_-10_) prior to filling in the questionnaire, and also at the age of 20 (W_20y_) [12]. We did not collect data on body composition as part of this study. At the final 6-year follow up examinations of surviving patients in 1995–1997, doctors reported the current antidiabetic treatment and the patients were weighed (W_6_) as above. Data from questionnaires were subject to meticulous control before and after they were entered into the database in order to ensure very high data quality. This was particularly important as data were used as feedback to the general practitioners in order for them to improve treatment quality.

### Definitions

Cardiovascular disease was defined as a history of myocardial infarction and/or history of stroke and/or angina pectoris and/or intermittent claudication and/or absent arterial pulses on both feet and/or amputation on the lower extremities. Peripheral neuropathy was defined as lack of a sense of pin prick and/or touch of cotton wool on at least one foot and/or absent patellar reflex on at least one knee. Diabetic retinopathy was defined as presence of at least one microaneurysm or further retinopathy. BMI_diag_ was calculated with height measured at diagnosis. Weight maintenance was defined as a weight change between +2 and -2 kg independent of length of follow up [13].

### Assays

Fasting blood samples were analysed at Odense University Hospital. The fraction of haemoglobin A1c (HbA1c) was determined by an ion-exchange HPLC protocol. The reference interval was 5.4–7.4%. Serum total cholesterols were measured enzymatically with cholesterol esterase-cholesterol oxidase-peroxidase reagent, and fasting serum triglycerides were determined enzymatically with a lipase-glycerolkinase-glycerol-3-phosphate oxidase-peroxidase reagent. Urinary albumin concentration was measured in freshly voided morning urine at Aarhus University Hospital by a polyethylene glycol radioimmunoassay.

### Statistical analysis

The influence of the weight history on weight development after diagnosis was analysed in three multivariate linear regression models. The dependent variable was the weight change between diagnosis and follow up (ΔW_diag/6_ = W_6_—W_diag_). The explanatory weight variables were: 1) W_20y_; 2) weight change from 10 years to 1 year before diabetes diagnosis (ΔW_-10/-1_ = W_-1_—W_-10_); and 3) weight change from 1 year before diabetes diagnosis until diagnosis (ΔW_-1/diag_ = W_diag_—W_-1_). The estimates of association for these three weight variables were adjusted for BMI_diag_ and the other baseline patient characteristics presented in Table 1.

**Table 1:** Patient characteristics.

Characteristics		Distribution or level	*n*
*At diabetes diagnosis*:			
Sex	Women	439 (49.6)	885
	Men	446 (50.4)	
Age (years)		63.2 (53.9; 71.4)	885
Body mass index (kg/m^2^) at diabetesdiagnosis, BMI_diag_		29.3 (26.3; 33.1)	880
Weight change (kg) from 10 years to 1 year before diabetes diagnosis, ΔW_-10/-1_		2.0 (-2.0; 10.0)	828
Weight change (kg) from 1 year before diabetes diagnosis till diagnosis, ΔW_-1/diag_		-1.5 (-5.5; 1.5)	868
Weight (kg) at age 20, W_20y_		65.5 (60.0; 75.0)	834
Living alone	Yes	254 (29.1)	872
	No	618 (70.9)	
Education	Basic school	668 (78.2)	854
	Higher	186 (21.8)	
Familial disposition to DM	No	474 (59.1)	802
	Yes	328 (40.9)	
Diagnostic plasma glucose (mmol/l)		13.8 (10.8; 17.0)	885
Haemoglobin A1c (fract., %) ^a^		0.099 (0.083; 0.115)	821
Fasting triglycerides (mmol/l)		2.1 (1.4; 3.0)	864
Total cholesterol (mmol/l)		6.3 (5.5; 7.2)	867
Urinary albumin (mg/l)		10.8 (5.6; 23.9)	850
Resting heart rate (beats/min)		76 (68; 84)	878
Systolic blood pressure (mmHg)		150 (130; 160)	880
Physical activity	Sedentary	206 (23.7)	870
	Active	664 (76.3)	
Smoking	Never	276 (31.7)	870
	Former	292 (33.6)	
	Current	302 (34.7)	
Cardiovascular disease	No	658 (75.6)	870
	Yes	212 (24.4)	
Diabetic retinopathy	No	780 (95.9)	813
	Yes	33 (4.1)	
Peripheral neuropathy	No	714 (81.9)	872
	Yes	158 (18.1)	
Cancer (former or present)	No	834 (95.6)	872
	Yes	38 (4.4)	
Allocation to type of care	Routine	421 (47.6)	885
	Structured	464 (52.4)	
*At 6-year follow up*:			
Antidiabetic treatment	Diet alone	264 (29.9)	884
	Oral agents	508 (57.5)	
	Insulin	112 (12.7)	

Limited to measurements from within 45 days of diabetes diagnosis. Reference range: 5.4–7.4%.

^a^ Values are numbers (%) or medians (interquartile range)

Because of multiple statistical testing, significance of the corresponding regression coefficient at the level of 0.01 was taken as evidence of a significant association with ΔW_diag/6_. In the multivariable analyses, the interaction with ΔW_-10/-1_, ΔW_-1/diag_, and W_20y_ respectively, was assessed for each covariate, and none of these terms were significant. For each interval-scale covariate, linearity was assessed by adding the corresponding quadratic term to the regression. None of these were significant. Simple comparisons were made with Kruskal-Wallis tests and χ^2^-tests. Data are freely available from the Research Unit of General Practice whose director may be contacted at feap@sund.ku.dk.

## Results

The 885 patients had baseline clinical characteristics typical of diabetes with hypertension, dyslipidaemia, and high prevalence of cardiovascular disease (Table 1). Medians (interquartile range (IQR), n) of the five weight measures, W_20y_, W_-10_, W_-1_, W_diag_, and W_6_, were 65.5 kg (60.0; 75.0, 834), 80.0 kg (72.0; 90.0, 828), 85.0 kg (75.0; 95.0, 868), 82.4 kg (72.0; 94.0, 885), and 80.0 kg (70.0; 91.1, 863), respectively. Thus most of the 15.5 kg median weight gain from age 20 until diagnosis had already occurred 10 years before diabetes diagnosis, and patients generally lost weight in the year preceding diagnosis as well as after diabetes diagnosis. BMI_diag_ was >25 kg/m^2^ for 83.5% of the men and 82.8% of the women in our study population. The median (IQR) length of the follow up period after diagnosis was 5.7 (5.1; 6.3) years. During this time period patients lost on average 2.5 (6.5; -1.7) kg.

While patients generally tended to lose weight during the 6 years after diabetes diagnosis, 35.2% (304/863) still gained weight. Similarly, although weight gain was common from 10 years to 1 year before diagnosis, 44.4% (368/828) actually lost weight, demonstrating that the weight changes both before and after diagnosis are characterised by great variability (S1 Table).

**Fig 2:**
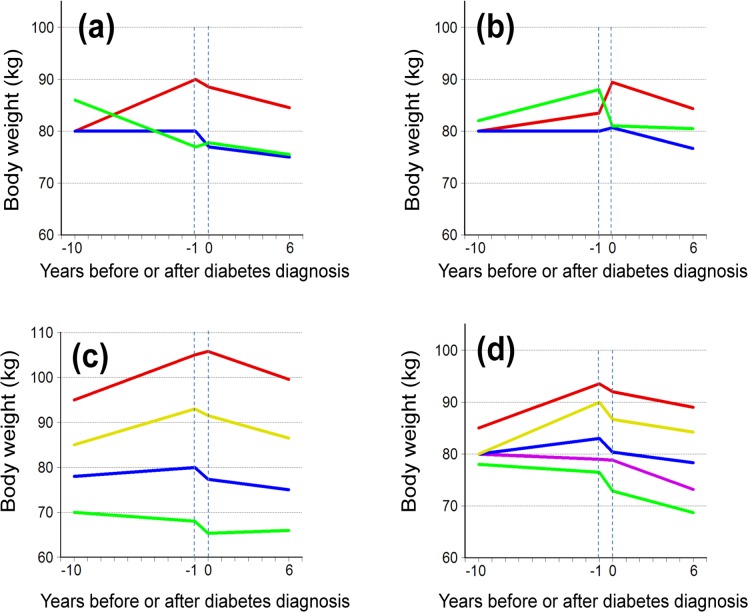
Weight changes around diabetes diagnosis according to weight history and diagnostic BMI and age. Weight changes from 10 years before to 6 years after diabetes diagnosis according to a: weight change (in kg) from 10 years to 1 year before diabetes diagnosis: weight loss >2 (green, n = 177); stable weight, i.e. weight change between +2 and -2 (blue, n = 239); and weight gain >2 (red, n = 412); b: weight change (in kg) from 1 year before diabetes diagnosis until diagnosis: weight loss >2 (green, n = 386); stable weight, i.e. weight change between +2 and -2 (blue, n = 300); and weight gain >2 (red, n = 182); c: body mass index (kg/m^2^) at diagnosis: <25 (green, n = 148); 25-<30 (blue, n = 336); 30-<35 (yellow, n = 265); ≥35 (red, n = 131); and d: age (years) at diagnosis: <50 (red, n = 143); 50-<60 (yellow, n = 220); 60-<70 (blue, n = 270); 70-<80 (lilac, n = 208); ≥80 (green, n = 44). The curves are defined by medians.

The predictive value of both W_20y_ and weight changes during the 10 years prior to diabetes diagnosis (ΔW_-10/-1_ and ΔW_-1/diag_) were analysed in three separate multivariable models (Table 2). The weight at age 20 and the weight change from 10 years to 1 year before diagnosis did not predict weight change after diagnosis (Fig 2a). However, each kg of weight gain during the year immediately before diagnosis was associated with a weight change (95% CI) of -0.20 kg (-0.28; -0.13, Table 2, Fig 2b) after 6 years. In two multivariable logistic regression analyses with predictor variables as above, but with the outcome ΔW_diag/6_ in three categories (weight loss > 2 kgs, weight gain > 2 kgs, and weight change between +2 and -2 kgs.), ΔW_-10/-1_ still did not predict weight change after diabetes diagnosis (*P* = 0.53, F-test) while ΔW_-1/diag_ still did (*P*<0.0001).

**Table 2:** Predictors of weight change during the first 6 years after diabetes diagnosis.

Patient characteristics at diabetes diagnosis		Model with weight change from 10 years to 1 year before diabetes diagnosis, ΔW_-10/-1_, *n* = 637		Model with weight change from 1 year before diabetes diagnosis until diagnosis, ΔW_-1/diag_. *n* = 665		Model with weight at age 20 years, W_20y_, *n* = 644	
		Estimate (95% CI)	*P*-value	Estimate (95% CI)	*P*-value	Estimate (95% CI)	*P*-value
Sex	Women	0		0		0	
	Men	-0.56 (-1.66; 0.54)	0.32	-0.71 (-1.77; 0.36)	0.19	-0.33 (-1.58; 0.91)	0.60
Age (10 years)		-1.47 (-2.02; -0.93)	<0.001	-1.42 (-1.94; -0.90)	<0.001	-1.47 (-2.01; -0.92)	<0.001
Body mass index (1 kg/m^2^) at diabetes diagnosis, BMI_diag_		-0.41 (-0.51; -0.30)	<0.001	-0.39 (-0.49; -0.29)	<0.001	-0.39 (-0.50; -0.29)	<0.001
Weight change (1 kg) from 10 years to 1 year before diabetes diagnosis, ΔW_-10/-1_		-0.01 (-0.06; 0.04)	0.79	-	-	-	-
Weight change (1 kg) from 1 year before diabetes diagnosis till diagnosis, ΔW_-1/diag_		-	-	-0.20 (-0.28; -0.13)	<0.001	-	-
Weight (1 kg) at age 20, W_20y_		-	-	-	-	-0.01 (-0.06; 0.03)	0.54
Living alone	Yes	0		0		0	
	No	-0.27 (-1.42; 0.88)	0.65	-0.42 (-1.52; 0.69)	0.46	-0.29 (-1.44; 0.85)	0.62
Education	Basic school	0		0		0	
	Higher	0.99 (-0.25; 2.23)	0.12	0.74 (-0.47; 1.95)	0.23	0.98 (-0.26; 2.22)	0.12
Familial disposition to DM	No	0		0		0	
	Yes	0.05 (-0.93; 1.03)	0.93	0.02 (-0.93; 0.97)	0.97	0.17 (-0.81; 1.15)	0.73
Diagnostic plasma glucose (1 mmol/l)		0.10 (0.02; 0.18)	0.020	0.07 (-0.01; 0.15)	0.092	0.11 (0.03; 0.19)	0.009
Fasting triglycerides (1 mmol/l)		0.16 (-0.04; 0.35)	0.11	0.12 (-0.08; 0.31)	0.24	0.15 (-0.05; 0.34)	0.14
Total cholesterol (1 mmol/l)		-0.37 (-0.78; 0.04)	0.077	-0.27 (-0.66; 0.12)	0.18	-0.38 (-0.79; 0.02)	0.063
Urinary albumin (10 mg/l)		0.00 (-0.02; 0.02)	0.98	0.00 (-0.02; 0.02)	0.86	0.00 (-0.02; 0.02)	1.00
Resting heart rate (10 beats/min)		0.20 (-0.24; 0.64)	0.37	-0.01 (-0.44; 0.41)	0.95	0.12 (-0.32; 0.55)	0.60
Systolic blood pressure (10 mmHg)		-0.09 (-0.34; 0.15)	0.46	-0.04 (-0.27; 0.20)	0.77	-0.11 (-0.35; 0.13)	0.38
Physical activity	Sedentary	0		0		0	
	Active	0.92 (-0.28; 2.13)	0.13	0.84 (-0.31; 2.00)	0.15	0.65 (-0.56; 1.85)	0.29
Smoking	Never	0		0		0	
	Former	1.43 (0.18; 2.68)	0.026	1.47 (0.26; 2.68)	0.017	1.18 (-0.07; 2.42)	0.065
	Current	-0.27 (-1.55; 1.02)	0.68	-0.56 (-1.80; 0.69)	0.38	-0.45 (-1.74; 0.83)	0.49
Cardiovascular disease	No	0		0		0	
	Yes	-1.03 (-2.26; 0.20)	0.10	-0.84 (-2.01; 0.33)	0.16	-1.07 (-2.28; 0.14)	0.082
Diabetic retinopathy	No	0		0		0	
	Yes	3.04 (0.25; 5.83)	0.033	2.83 (0.17; 5.50)	0.037	3.53 (0.67; 6.38)	0.016
Peripheral neuropathy	No	0		0		0	
	Yes	-0.33 (-1.64; 0.98)	0.62	-0.28 (-1.54; 0.97)	0.66	-0.26 (-1.56; 1.04)	0.69
Cancer (former or present)	No	0		0		0	
	Yes	0.68 (-1.72; 3.07)	0.58	0.67 (-1.57; 2.91)	0.56	0.61 (-1.67; 2.89)	0.60
Allocation to type of care	Routine	0		0		0	
	Structured	-1.00 (-1.98; -0.01)	0.047	-1.03 (-1.98; -0.08)	0.033	-0.98 (-1.95; 0.00)	0.050

Values are estimates (95% confidence intervals) from 3 multivariable linear regression models. P-values are from the corresponding t-tests. The dependent variable is the change in weight (ΔW_diag/6_) from diabetes diagnosis until the final follow up examination on average 5.7 years later. Patient characteristics are explanatory variables. The estimate can be interpreted as the difference in weight change from diagnosis until 6-year follow up for every unit change (or relative to reference category) of the predictor variable in question.

### Multivariable analyses

**Fig 3:**
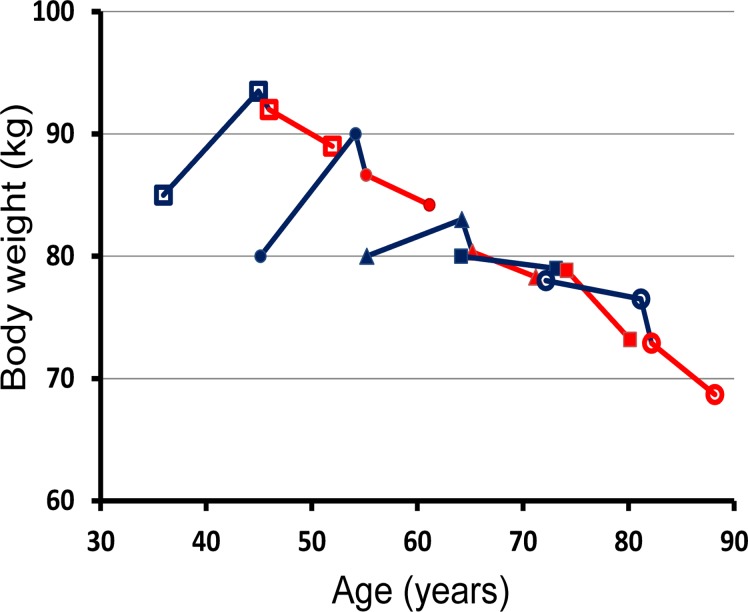
Overview of age-related weight developments. Weight changes from 10 years before to 6 years after diabetes diagnosis according to age at diagnosis (years): 40–49 (open squares, n = 143); 50–59 (filled circles, n = 220); 60–69 (filled triangles, n = 270); 70–79 (filled squares, n = 208); 80+ (open circles, n = 44). The curves which are defined by medians are blue before diagnosis and red after diagnosis.

BMI_diag_ and age were also important predictors of ΔW_diag/6_ (Table 2). As expected, the higher the BMI_diag_, the greater the weight loss after diagnosis (Fig 2c). The age-related differences in weight reduction after diagnosis were modest and barely distinguishable in Fig 2d, but the influence of age on the change in the weight developments in connection with diabetes diagnosis is considerable (Fig 3).

In comparison with the 885 patients in the present study sample, the 389 patients who died or dropped out before 6-year follow up had a male preponderance (57.8% vs. 50.4%, *P* = 0.014), were older (70.1 years vs. 63.2 years, *P*<0.001), had lower BMI (28.3 kg/m^2^ vs. 29.3 kg/m^2^, *P* = 0.008), and had smaller ΔW_-10/-1_ (0 (-3; 7) kg vs. 2 (-2; 10) kg, medians (IQR), *P* = 0.011). There was no difference between the two groups regarding diagnostic plasma glucose (*P* = 0.96), systolic blood pressure (*P* = 0.21), W_20y_ (*P* = 0.056), and ΔW_-1/diag_ (*P* = 0.45).

## Discussion

In accordance with previous findings among Pima Indians [7], this study found that Danish patients with type 2 diabetes tend to gain weight until shortly before their diabetes is diagnosed, and progressively lose weight afterwards. Indeed, six years after diabetes diagnosis, patients with type 2 diabetes maintained an average weight loss of 2.5 kg. This weight loss was unrelated to long-term weight changes before diabetes diagnosis, when age, sex, BMI, and other patient characteristics were accounted for (Table 2). The general weight loss from diagnosis until 6-year follow up was primarily explained by increasing age, with approximately 1.5 kg weight loss per extra decade, and by higher BMI at diagnosis, with approximately 0.4 kg per unit increase in BMI. A weight loss after diabetes diagnosis seems to be a realistic option both when rapid weight gain has taken place just before diagnosis (Fig 2b), and when a long-term weight gain has preceded diagnosis (Fig 2a). The weight loss after diagnosis seems to be dependent on treatment intensity to a minor degree, as approximately 1 kg of this weight loss in the present analysis was attributed to being allocated to the intervention arm of the trial (Table 2). Without access to a measure of body composition, we were not able to estimate the degree to which weight loss consisted of lean body mass or fat.

Until now, weight changes before and after diabetes diagnosis have only been analysed together in Pima Indians, in whom an average weight gain before diagnosis was followed by a weight loss of similar size after diagnosis, at least in the long run [7]. The weight gain seemed to continue in the first 1–2 years after diabetes diagnosis, even if this observation is most likely dependent on infrequent data sampling. The DCGP study provides detailed and valid information about the change in weight developments in connection with diabetes diagnosis in a population-based, homogenous sample of Danish patients with type 2 diabetes. Six years after diabetes diagnosis the quality of the glycaemic control of our patients was on a level with that in e.g. UKPDS [14], although fewer of the patients in the DCGP study received antidiabetic pharmacological treatment than in UKPDS [8]. This happened in spite of a very high level of diagnostic plasma glucose in the DCGP study [8], and it is likely that the relative benignity of the weight developments in this study contributed to this result [9].

### Weight developments before diabetes diagnosis

Given the measuring points in our study, the main historic weight gain before diabetes diagnosis took place sometime between the age of 20 and the average age of 53.2 years, 10 years before diabetes diagnosis, equivalent to 0.44 kg/year on average (14.5 kg/33.2 years). In the 9-year period up to one year before diagnosis the weight gain rate was 0.56 kg/year (5 kg/9 years), although this average value is hiding a sizable age-related variation (Fig 2c). These rates are considerably larger than the 0.16 kg/year until the age of 65 years reported in men unselected for disease [15]. Thus, despite some individual variation, the development of diabetes was not preceded by a recent weight gain, but rather by a continuous, larger than normal, weight gain over several decades, at least up until the age of about 60 years (Fig 3).

Near-diagnostic weight history has only been touched upon a few times in previous literature [7,16]. In the present study the average weight loss in the year just before diagnosis was 2.6 kg, and appeared at almost all ages, but not in the most obese (Fig 2c and 2d). Type 2 diabetes may go unnoticed for a long time, and at diagnosis, 35% of the patients in the DCGP study reported having experienced unintended weight loss, usually lasting for less than 6 months [17]. Both the prevalence of this indicator of disease severity and the size of the average pre-diagnostic weight loss increased with diagnostic plasma glucose [16,17]. This supports the view that pre-diagnostic weight loss may be explained by energy loss in glycosuria, which was present in 43% of our patients at diagnosis [18].

This weight development in the year prior to diabetes diagnosis is almost certainly driven by the decreasing anabolic influence from insulin following a progressive burn-out of the beta-cells, but it is an open question whether or not the huge general weight gain in the preceding 9 years or more is also influenced by disease processes. Dysfunction of the beta-cell secretion pattern is present long before glucose levels rise into pre-diabetes [19], and it is an ongoing discussion, based on data from adolescents, whether type 2 diabetes is caused by weight gain, or whether weight changes are induced by the changing size and pattern of insulin secretion [20,21].

While there is little doubt that carrying extra weight and obesity cause insulin resistance, and that a high BMI is associated with an increasing risk of developing diabetes [22], the evidence is conflicting on whether insulin secretion rises to compensate for insulin resistance, or whether high insulin secretion promotes body weight gain and the subsequent development of insulin resistance [20,21].

### The association of weight changes before and after diabetes diagnosis

Short-term weight changes in one direction are usually followed by a weight change in the opposite direction [23]. This common weight trend helps to explain why weight changes in the year immediately before diabetes diagnosis predict the weight changes after diagnosis (Fig 2b), and it demonstrates the importance of distinguishing recent from distant weight history. Regression towards the mean may contribute, but only to the extent of explaining the relation between recent weight history and weight changes thereafter.

Several studies have cautioned the use of recalled weights particularly for evaluation of individual patients, whereas most find that in surveys, self-reported weights often appear acceptable and come close to the assessed population mean [24,25]. In our study, we cannot exclude, that the observed difference in the impact of distant and recent weight history may be due to differential recall bias. Indeed, our own validation study clearly suggested that while patients recalled their 1-year body weight with no average bias, the 10-year recalls introduced an underestimation of the measured weight by approximately 2 kg [12]. It may be argued that weight recall biases are rarely random and recalls often depend on current weight status. However, in our own weight validation study, the validity of patients’ recall was independent of age, sex, and also current weight status, in contrast to other studies [25,26]. Of course, we cannot exclude the possibility that the imprecision and error in the recalled weights may still be associated with the later weight changes after diagnosis. However, in general such measurement errors will tend to attenuate the effects of the weight history variables on later weight changes, and hence they are not expected to invalidate the present findings. The attained weight at diagnosis may only be ascribed to treatment to a minor degree, as the majority of patients had their body weight measured soon after diagnosis [16].

### Weight developments after diabetes diagnosis

Especially when newly diagnosed, diabetes may serve as a spur to action for patients and probably doctors too [6,27]. It appears from Fig 3 that average body weight after diagnosis follows a common trajectory of decline. In the background population, average body weight seems to increase until the age of 50–65 years [15] and then plateaus or starts a slow decline together with loss of muscle mass and beta-cell mass. At the same time, waist circumference, abdominal fat and insulin resistance continue to increase [28], thus creating the balance that means when older patients with a long-term weight loss are diagnosed with diabetes, they continue to lose weight after diagnosis (Fig 3). For example, in the Look AHEAD trial the oldest participants lost relatively more weight than the younger ones [29]. A cohort effect of an underlying increasing average body weight in the background population, especially among the young [15], may have made this age-related average weight loss after diagnosis steeper than anticipated from age alone.

### Further strengths and limitations of the study

The list system in Denmark with its well-defined background population in all the participating general practices, the constant inclusion activity over time, and the few primary exclusions [8], all suggest that our patients are likely to be representative of all patients with newly diagnosed, clinical, and often symptomatic diabetes in this age group. Unlike most other trials, the DCGP study did not have an upper age limit for participants, and the wide age range enabled us to study age-related differences in weight changes with many details. However, the present study also has further limitations. For instance, follow up studies indicate that muscle mass decreases approx. 6% per decade when it has reached its peak around the age of 45 [30]. In the DCGP study the amount of lean tissue lost most certainly differed between relatively young and older patients, but we did not have access to a measure of body composition. Instead we have given a detailed account of the overly strong influence of age on body weight developments before and after diabetes diagnosis. Another limitation is that our data come from a randomised trial, but it is unlikely that the observed relationships are results of the intervention as this only had a small influence on weight 6 years after diagnosis (Table 2) [8]. Also, the patients in this study presented with clinical diabetes, and the results cannot be generalized directly to diabetes patients diagnosed nowadays where case-finding is much more common. Finally, we did not analyse weight after diagnosis as a time-dependent variable as we have detailed follow up data only from the intervention group. Neither were covariates from follow up included, which e. g. could contribute to the lack of association between level of physical activity and weight loss during 6 years after diagnosis (Table 2).

### Implications for clinical practice

In the DCGP study an average weight loss of approximately 2.5 kg was maintained 6 years after diagnosis in spite of the similarly sized average weight loss immediately before diabetes diagnosis. This result is indeed in contrast to the well-known, seemingly inexorable, weight gain after diabetes diagnosis observed in major diabetes trials [14,31–34], but more importantly, it is in accordance with a similar weight loss observed during the first 5 years after diabetes diagnosis in the ADDITION-Europe study [27]. In this latter randomized intervention study, newly diagnosed patients were screen-detected and the weight loss was independent of treatment allocation, as in the DCGP study [8]. This tendency to succeed with a lasting weight loss after diagnosis may reflect what is happening in Western-European primary care [9], both when patients are treated intensively to target [27], and when treatment targets are individualised [8]. Observational studies also indicate that weight changes in diabetes treatment are not all uphill [35], and in the Look AHEAD study the intervention group maintained a weight loss of approximately 3 kg compared to the control group after 6 years of intervention [36].

Inspiration for weight control may also be found in the Diabetes Prevention Program, where intensive, individualised lifestyle counselling was more effective in reducing risk of diabetes than metformin [37], and the risk reduction with lifestyle modification was closely related to reduced body size and fat, whereas it was independent of these changes in the metformin arm.

In conclusion, during the first 6 years after diagnosis, type 2 diabetic patients follow a trajectory of declining average weight, and these weight developments are related primarily to recent, rather than to long-term historic weight changes. Furthermore, this pattern is seen irrespective of BMI and age at diagnosis. The diabetes diagnosis seems to be a window of opportunity for obtaining a lasting weight loss irrespective of previous long-term weight changes.

## Supporting Information

S1 Table Characteristics of patients who gained or lost weight before or after diabetes diagnosis (DOCX)
