# Supplementary material for: Weight Changes following the Diagnosis of Type 2 Diabetes: The Impact of Recent and Past Weight History before Diagnosis. Results from the Danish Diabetes Care in General Practice (DCGP) Study
Source: PLoS One. 2015 Apr 15;10(4):e0122219. doi: 10.1371/journal.pone.0122219 (PMC4398495; doi:10.1371/journal.pone.0122219)
Supplement: S1 Table — (DOCX) [file pone.0122219.s001.docx]

**S1 Table. Characteristics of patients who gained or lost weight before or after diabetes diagnosis**

| Characteristics |  | Weight change from 10 years to 1 year before diabetes diagnosis | | | Weight change from diabetes diagnosis until 6 years later | | |
| --- | --- | --- | --- | --- | --- | --- | --- |
|  |  | Weight gain | Weight loss | *P*-value | Weight gain | Weight loss | *P*-  value |
| *At diabetes diagnosis:* |  |  |  |  |  |  |  |
| Sex | Women | 214 (52.6) | 193 (47.4) | - | 139 (32.7) | 286 (67.3) | - |
|  | Men | 246 (58.4) | 175 (41.6) | 0.094 | 165 (37.7) | 273 (62.3) | 0.13 |
| Age (years) |  | 60.7 (53.2; 69.0) | 66.2 (56.3; 73.7) | <0.001 | 60.8 (52.4; 67.4) | 64.1 (54.7; 72.1) | <0.001 |
| Body mass index (kg/m^2^) at diabetes diagnosis, BMI_diag_ |  | 30.5 (27.4; 34.2) | 28.0 (25.0; 31.5) | <0.001 | 27.6 (24.7; 31.0) | 30.3 (27.3; 33.8) | <0.001 |
| Weight change (kg) from 10 years to 1 year before diabetes diagnosis, ΔW_-10/-1_ |  | 8 (5; 14) | -2 (-7; 0) | <0.001 | 2 (-2; 8) | 3 (-2; 10) | 0.047 |
| Weight change (kg) from 1 year before diabetes diagnosis till diagnosis, ΔW_-1/diag_ |  | -1.8 (-6.2; 1.6) | -1.4 (-5.0; 1.4) | 0.52 | -3.5 (-8.3; 0.5) | -0.7 (-4.0; 2.0) | <0.001 |
| Weight (kg) at age 20, W_20y_ |  | 66 (60 ; 75) | 67 (60; 75) | 0.24 | 65 (60; 75) | 67 (60; 75) | 0.22 |
| Living alone | Yes | 120 (50.0) | 120 (50.0) | - | 89 (35.5) | 162 (64.5) |  |
|  | No | 340 (57.9) | 247 (42.1) | 0.045 | 210 (35.1) | 389 (64.9) | 0.94 |
| Education | Basic school | 340 (53.0) | 290 (46.0) | - | 217 (33.2) | 436 (66.8) |  |
|  | Higher | 113 (62.4) | 68 (37.6) | 0.051 | 78 (43.3) | 102 (56.7) | 0.014 |
| Familial disposition to DM | No | 257 (57.2) | 192 (42.8) | - | 154 (33.7) | 303 (66.3) |  |
|  | Yes | 167 (53.2) | 147 (46.8) | 0.30 | 117 (36.1) | 207 (63.9) | 0.49 |
| Diagnostic plasma glucose (mmol/l) |  | 13.5 (10.7; 17.0) | 13.8 (10.8; 16.9) | 0.60 | 14.7 (11.1; 18.1) | 13.5 (10.6; 16.6) | 0.044 |
| Haemoglobin A1c (fract., %)  ^a^ |  | 9.7 (8.2; 11.2) | 10.1 (8.4; 11.7) | 0.055 | 10.0 (8.4; 12.0) | 9.8 (8.3; 11.2) | 0.091 |
| Fasting triglycerides (mmol/l) |  | 2.1 (1.4; 3.2) | 2.0 (1.4; 2.8) | 0.003 | 2.0 (1.3; 2.8) | 2.0 (1.5; 3.0) | 0.48 |
| Total cholesterol (mmol/l) |  | 6.3 (5.5; 7.2) | 6.3 (5.5; 7.2) | 0.22 | 6.3 (5.5; 7.2) | 6.3 (5.5; 7.2) | 0.98 |
| Urinary albumin (mg/l) |  | 10.6 (5.5; 21.3) | 10.7 (5.5; 27.5) | 0.46 | 11.3 (6.3; 23.2) | 10.5 (5.3; 24.9) | 0.26 |
| Resting heart rate (beats/min) |  | 76 (68; 82) | 76 (68; 84) | 0.74 | 76 (68; 84) | 76 (68; 84) | 0.14 |
| Systolic blood pressure (mmHg) |  | 145 (130; 160) | 150 (135; 165) | 0.015 | 140 (130; 160) | 150 (135; 160) | <0.001 |
| Physical activity | Sedentary | 111 (58.4) | 79 (41.6) | - | 57 (28.6) | 142 (71.4) | - |
|  | Active | 347 (54.7) | 288 (45.3) | 0.41 | 242 (37.3) | 407 (62.7) | 0.028 |
| Smoking | Never | 122 (47.5) | 135 (52.5) | - | 83 (30.7) | 187 (69.3) | - |
|  | Former | 184 (65.5) | 97 (34.5) | - | 110 (38.7) | 174 (61.3) | - |
|  | Current | 154 (53.7) | 133 (46.3) | 0.001 | 106 (36.0) | 188 (63.0) | 0.14 |
| Cardiovascular disease | No | 361 (57.2) | 270 (42.8) | - | 242 (37.4) | 405 (62.6) | - |
|  | Yes | 97 (50.3) | 96 (49.7) | 0.098 | 58 (28.7) | 144 (71.3) | 0.028 |
| Diabetic retinopathy | No | 416 (56.7) | 318 (43.3) | - | 265 (34.7) | 498 (65.3) | - |
|  | Yes | 11 (35.5) | 20 (64.5) | 0.026 | 17 (53.1) | 15 (46.9) | 0.039 |
| Peripheral neuropathy | No | 370 (54.9) | 304 (45.1) | - | 248 (35.7) | 447 (64.3) | - |
|  | Yes | 82 (58.2) | 59 (41.8) | 0.52 | 52 (33.3) | 104 (66.7) | 0.64 |
| Cancer (former or present) | No | 443 (55.9) | 349 (44.1) | - | 284 (34.9) | 529 (65.1) | - |
|  | Yes | 17 (48.6) | 18 (51.4) | 0.39 | 15 (40.5) | 22 (59.5) | 0.49 |
| Allocation to type of care | Routine | 217 (55.2) | 176 (44.8) | - | 161 (39.3) | 249 (60.7) | - |
|  | Structured | 243 (55.9) | 192 (44.1) | 0.89 | 143 (31.6) | 310 (68.4) | 0.019 |
| *At 6-year follow-up:* |  |  |  |  |  |  |  |
| Antidiabetic treatment | Diet alone | 147 (58.8) | 103 (41.2) | - | 63 (24.7) | 192 (75.3) | - |
|  | Oral agents | 267 (56.1) | 209 (43.9) | - | 181 (36.3) | 318 (63.7) | - |
|  | Insulin | 45 (44.6) | 56 (55.4) | 0.048 | 60 (45.0) | 49 (55.0) | <0.001 |

Values are numbers (%) or medians (interquartile range)

^a^ Limited to measurements from within 45 days of diabetes diagnosis. Reference range: 5.4-7.4%.
